# Flow-cytometry Assessment of DNA content and Immunophenotyping of Immune-cells in Lymph-node-specimens as a Potential Diagnostic Signature of Aggressiveness in B-Non-Hodgkin Lymphomas

**DOI:** 10.1007/s00277-024-05807-8

**Published:** 2024-05-23

**Authors:** David Azoulay, Tal Tapuchi, Ohad Ronen, Luiza Akria, Hector I. Cohen, Celia Surio, Svetlana Rodin Chepa, Elizabeth Eshel, Moran Zarfati, Galia Stemer, Netanel A. Horowitz

**Affiliations:** 1 Azrieli Faculty of Medicine, Bar Ilan University, Safed, Israel; 2 Department of Otolaryngology – Head and Neck Surgery, Director Head and Neck Surgery Unit, Galilee Medical Center, Nahariya, Israel; 3 Hematology Unit and Laboratories, Galilee Medical Center, Nahariya, Israel; 4 Pathology Unit and Laboratories, Galilee Medical Center, Nahariya, Israel; 5 Hematology Unit and Laboratories, Ziv Medical Center, Safed, Israel; 6 The Ruth and Bruce Rappaport Faculty of Medicine, Department of Hematology and BMT, Rambam Health Care Campus, Israel Institute of Technology, Haifa, TechnionHaifa, Israel

**Keywords:** Lymph, Node (LN), Aggrressive, Lymphoma, Indolent, Lymphoma, DNA, Cell, Cycle, Indexing, immune, Cells, tumor, Microenvironment

## Abstract

Flow-cytometry (FC) is a powerful tool that can assist in lymphoma diagnosis in lymph node (LN) specimens. Although lymphoma diagnosis and classification are mainly based on tumor cell characteristics, surrounding cells are less employed in this process. We retrospectively investigated alterations in the ploidy status, proliferative cell fraction (PF) and the percentages of surrounding immune cells in 62 consecutive LN specimens with B-Cell Non-Hodgkin Lymphoma (B-NHL) that were submitted for FC evaluation between 2019–2022. Compared with indolent B-NHLs, aggressive B-NHLs show increased DNA aneuploidy and PF, increased monocytes, immature-granulocytes, mature granulocytes, CD8^+^ T-cells, Double-Negative-T-cells and Double-Positive-T-cells, and decreased total CD45^+^ cells, total lymphocytes, CD4^+^ T-cells and CD4/CD8 ratio. Receiver operating characteristic analysis determined PF > 6.8% and immature-granulocytes > 0.9% as optimal cutoffs with highest specificity and sensitivity in differentiating aggressive and indolent B-NHLs. These findings further strength the diagnostic value of DNA content analysis by FC and suggest the utilization of tumor surrounding immune cells in NHL diagnosis and classification.

**Supplementary Information:**

The online version contains supplementary material available at 10.1007/s00277-024-05807-8.

## Introduction

Flow cytometry (FC) is a diagnostic tool used for rapid multi parameters analysis of liquid suspended cells that is worldwide used in clinical diagnostic laboratories. This method allows rapid analysis of multiple characteristics in a large number of cells in a short time [1]. Cell analysis using FC is based on physical characters that include relative cell size and complexity, and on fluorescent-conjugated-antibody based detection of intracellular or cell surface proteins termed Cluster of Differentiation (CDs) [2]. In addition to CDs, the use of chemical markers for DNA analysis such as propidium iodide (PI) or DRAQ5, could add important information on the cell cycle including the proliferation and apoptosis status of the cells [1, 2].

FC has a tremendous contribution to the diagnosis and classification of hematological malignancies such as leukemia and lymphoma [3]. It has a central role not only in the identification and quantification of malignant cells in the specimens, but it also adds crucial information needed for the classification and differential diagnosis of leukemia and lymphomas subtypes [4]. Peripheral blood and bone marrow aspirates are naturally suitable specimens,routinely screened in most clinical FC laboratories. In certain laboratories including ours, cell suspension is also prepared from lymph-node (LN) biopsies or aspirates and routinely examined for lymphomatoid malignancies as part of the diagnostic process.

B-Cell-Non-Hodgkin's-Lymphomas (B-NHLs) comprise the largest group of lymphomas in the western world. When using FC for B-NHLs diagnosis in a LN, the tumor B-cells are usually identified by their immunoglobulin (Ig) light-chain restriction and by aberrant markers expression profile. These tumor B-cells are typically detected in the background of inflammatory lymphoid and myeloid derived immune cells that could be analyzed and characterized simultaneously by the same method. Hence, in this clinical scenario, FC is an attractive tool as it can simultaneously detect and classify the Ig restricted lymphoma cells and characterize them in the mixed populations of remaining healthy cells [5]. This advantage becomes more evident in minimally invasive biopsies where there is little biologic material, and the tissue architecture is not always preserved for performing an intact histological evaluation.

The biological features and clinical behavior of B-NHLs range from indolent (e.g., grade 1 Follicular lymphoma) to aggressive entities (e.g., Diffused Large B cell lymphoma or Burkitt cell lymphoma). The exact classification of a B-NHL subtype is very important for precision therapy. However, this process is not always clear when tissue biopsy is not available, inadequate, or not representative. In addition, in some B-NHLs within the same classification such as in mantle cell lymphoma (MCL) the disease behavior could range between indolent to aggressive [3], suggesting a spectrum of disease agressivness rather than a fixed point. Furthermore, as learned from the transformation of FL to Diffused-Large-B-cell-lymphoma (DLBCL), indolence seems to be a dynamic process that could change with the disease course and between different malignant sites in the same patient [4]. Therefore, defining FC based measurable biomarkers that could help in fast and accurate monitoring of lymphoma subtype is essential.

Our laboratory has implemented DNA content analysis by propidium iodide (PI), as part of the routine FC diagnostic workup. In a previous publication we demonstrated the applicability of S-phase and proliferating cell fraction (PF) determination by FC as a tool for differentiation between aggressive and indolent CD10 positive B-NHLs [6]. In the current study we aim to focus on LN biopsies and extend our previous observations to include specimens of CD10 positive and negative B-NHLs. Specifically, we would like to characterize differences in cell cycle parameters and relative incidences of surrounding immune cells in B-NHLs and to test their utility in discriminating between indolent and aggressive B-NHLs.

## Materials and methods

### Case selection

This is a retrospective analysis of FC data on consecutive LN specimens obtained from patients who were presented with lymphadenopathy and underwent a diagnostic biopsy at the Galilee Medical Center (Nahariya, IL) between the years 2019–2022. The FC analysis was carried out on single cell suspension prepared from fresh, LN tissue or aspirate specimens. Only specimens with a definitive histologic diagnosis of B-NHL, established by well trained hematopathologist were included in the study. The specimens were divided into aggressive and indolent according to the accepted WHO criteria and their pathological record (the classification and prevalence of the B-NHLs in our study are summarized in supplementary Table 1). Specimens with T-cell or Hodgkins lymphoma and non-hematological malignancies were excluded from the current study. Specimens with inadequete cells for FC analysis (i.e. mistakenly placed in preservative) or specimens with incomplete FC data were excluded as well.

### FC analysis

FC was performed on fresh specimens that were collected in 0.9% NaCl solution (B. Braun Melsungen, Germany) without any preservative. Tissue biopsies were mechanically processed into a single cell suspension in phosphate buffer saline (PBS) or RPMI 1640 (Biological Industries, Beit-Haemek LTD, Israel) within 24 h of isolation, and washed and suspended in PBS before staining. In case of aspirations from the LN area, the sample was centrifuged, and cell pellet was suspended in PBS before staining. For staining, samples of 50 µL cell suspension (containing approximately 1X10^4^—1X10^5^ cells) were placed in a separated polypropylene FACS tubes. For DNA cell cycle analysis, the cells immediately stained for DNA content, using a Coulter DNA prep REAGENT Kit ^5^ according to the manufacturer's instructions. Briefly, 50 µL of reagent containing detergent were added and vortexed for 15 s. Then, reagent containing the dye and the red cell lysing solution was added and vortexed for 8 s. Samples were read using a Beckman Coulter Navios flow cytometer instrument using the FL3 channel and selection of singlet events was done using FL4 peak against FL3 and FL4 time of flight (TOF) against FL3. Peripheral blood leukocytes of healthy donors were used as a calibration standard to determine the G_0_/G_1_ peak of DNA diploid with X-median of approximately 200 in a linear scale. DNA index (DI) as well as the estimation of cells in S and G_2_M cell cycle compartments (Proliferative Fraction) were performed by an expert manual gating analysis. For immune cell population screening we added 7 µL of our lymphocyte screening antibody cocktail containing; CD7 Pacific-Blue, CD45 PE-Cy7, CD56 PE-Cy5, CD3 ECD, CD64 + CD8 PE and CD19 + CD4 FITC (All mAb from Beckman Coulter Inc. Brea CA) into a new sample of 50 µL cell suspension. After 10 min incubation at room temperature protected from light, the samples were washed and suspended in 500 µL PBS. The samples were acquired on the flow cytometer and at least 20,000 nucleated CD45^+^ cells were recorded. Using CD45/SSC gating strategy, we determined and recorded the percentages of: Total CD45^+^ cell component within the total nucleated cells, the percentages of lymphocytes (CD45^+^/SSC^low^), Monocytes (CD45^+^/CD64^high^/SSC^dim^), Mature Granulocytes (CD45^+^/CD64^low^/SSC^high^) and Immature Granulocytes (CD45^+^/CD64^high^/SSC^high^) within the total CD45^+^ cell component. We also determined and recorded the percentages of B-cells (CD19^+^), T-cells (CD3^+^) and NK cells (CD3^−^/CD56^+^) within the total lymphocytes and the percentages of CD4^+^ T cells, CD8^+^ T cells, CD4^+^/CD8^+^ double positive T-cells (DPT), CD4^−^/CD8^−^ double negative T-cells (DNT) and CD3^+^/CD56^+^ NK-T cells within the total T-cells.

### Statistical analysis

The percentages of cells in proliferative fraction (PF) and the percentages of all immune cell populations was assesed by using T-test for comparing 2 independent variables or ANOVA test for multivariable comparison. The Pearson chi-square analysis was used to compare non-parametric variables between groups. A Receiver Operating Characteristic (ROC) analysis was generated and the area under the curve was calculated to evaluate the optimal cutoffs of the variables between indolent and aggressive lymphomas, with the highest degree of sensitivity and specificity. All statistical analyses were performed using JMP (SAS Inc.) statistical software.

## Results

1. Differences between specimens with aggressive B-NHLs and indolent B-NHLs

**Fig. 1:**
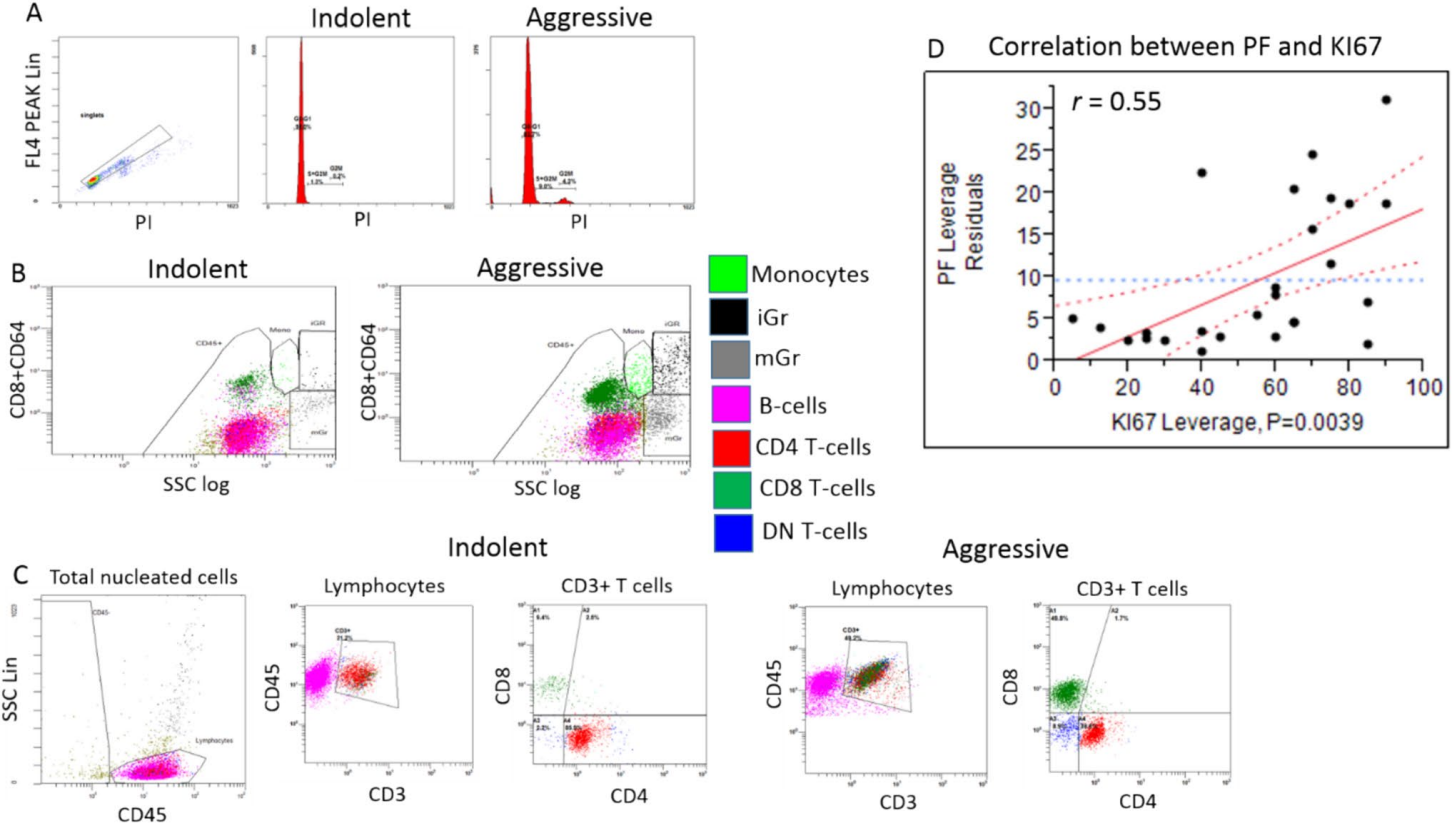
Gating strategy and representative plots of indolent and aggressive specimens. A. Representative histograms of DNA content analysis. Left plot show the gating strategy for excluding debris from the DNA content analysis as lymphoma samples frequently contain significant debris underlying all phases of the cell cycle. B. Total CD45^+^ cell component within the total nucleated cells (total CD45^+^) and the percentages of lymphocytes (CD45^+^/SSC^low^) within the total CD45^+^ cell component that were determined using CD45 vs. lin SSC gating strategy. Representative plots showing the gating strategy for determining the level of Monocytes (CD45^+^/CD64^high^/SSC^dim^), Mature Granulocytes (CD45^+^/CD64^low^/SSC^high^) and Immature Granulocytes (CD45^+^/CD64^high^/SSC^high^) (marked by the light green, black and gray color respectively) within the total CD45^+^ cell component. C. The percentages of total lymphocytes were determined using CD45 vs. lin SSC gating strategy after excluding the CD45 negative cell events. T-cells (CD3^+^), B cells (CD19^+^) (marked by pink color) and NK cells (CD3^−^/CD56^+^) (not shown) were determined within the total lymphocytes in the specimens. Representative plots showing the gating strategy for determining the percentages of CD4^+^ T cells (marked by red color), CD8^+^ T cells (marked by dark green color) and CD4^−^/CD8^−^ double negative T-cells (DNT) (marked by blue color) within the total T-cells. D. Correlation (*r* = 0.55) between PF as determined by flow cytometry in the specimens, and KI67 as determined in the tissue by immunohystochemistry

Histopathological analysis determined B-Cell-Non-Hodgkin's-Lymphoma (B-NHL) in 62 specimens. The gender and the age in years (F:M; median and range) of the patients from which the specimens were isolated were 34:28 and 65.65 and 27.4—80. The distribution of the type of specimens (tissue/aspirate, n and %) were 41:21 and 66:34 respectively. Regarding DNA content analysis parameters, specimens with aggressive B-NHLs demonstrated a high rate of DNA-aneuploidy compared to indolent B-NHLs. Aggressive B-NHLs specimens were found to have significantly higher percentages of cells in PF relative to indolent B-NHLs. The levels of cells in PF show positive correlation with the proliferative index determined by ki-67 staining, in the lymph node biopsies (Fig. 1 D). Regarding to immune cells, the percentages of CD45^+^ cells of the total nucleated cells and the percentages of lymphocytes of the CD45^+^ cells were lower in the aggressive B-NHLs specimens compared to the indolent B-NHLs (*p value* = 0.001). The percentages of Monocytes, mature Granulocytes (mGr) and iGr of the CD45^+^ cells were significantly higher in the aggressive B-NHLs specimens compared to the indolent B-NHLs (*p value* = 0.0005, 0.018 and 0.0001 respectively). The percentages of CD8 T-cells, DP T-cells and DN T-cells were significantly higher in aggressive B-NHLs specimens compared to the indolent B-NHLs (*p value* = 0.0002, 0.002 and 0.0004 respectively). The percentages of CD4 T-cells and the CD4/CD8 ratio were significantly lower in aggressive B-NHLs specimens compared to the indolent B-NHLs (*p value* = 0.002 and 0.0005 respectively) (Gating strategy and representative plots of indolent and aggressive specimens are shown in Fig. 1 A-C, differences between specimens with aggressive and indolent B-NHLs are summarized in Table 1).

2. Percentages of cells in PF and iGr show potential to differentiate specimens with aggressive and indolent B-NHL with high specificity and sensitivity

**Table 1:** Differences between specimens with aggressive and indolent B-NHLs

**Parameter**	**Aggressive **	**Indolent**	P value Aggressive vs. Indolent
Ploidy % Diploid/Uneuploid	51.5: 48.5	89:11	0.002
PF (% of total cells)	12.85 ± 9.35	4.08 ± 2.39	<0.0001
Total CD45^+^ cells (% of nucleated cells)	88.13 ± 12.41	94.85 ± 8.32	0.019
Lymphocytes (% of total CD45^+^ cells)	74.35 ± 23.08	90.88 ± 11.22	0.001
Monocytes (% of total CD45^+^ cells)	4.13 ± 4.32	1.03 ± 0.74	0.0005
mGr (% of total CD45^+^ cells)	9.57 ± 14.69	2.58 ± 2.78	0.0180
iGr (% of total CD45^+^ cells)	4.23 ± 4.07	0.93 ± 0.89	0.0001
B-cells (% of total lymphocytes)	54.28 ± 24.65	60.28 ± 18.38	0.307
NK-cells (% of total lymphocytes)	1.01 ± 1.87	0.46 ± 0.42	0.137
T-cells (% of total lymphocytes)	40.42 ± 21.77	36.43 ± 15.76	0.429
CD4 (% of T cells)	64.32 ± 15.40	78.04 ± 9.97	0.0002
CD8 (% of T cells)	35.07 ± 16.00	20.75 ± 10.53	0.0002
CD4/CD8 ratio	2.61 ± 2.09	4.49 ± 1.79	0.0005
DP T (% of T cells)	7.04 ± 5.93	3.21 ± 2.05	0.002
DN T (% of T cells)	6.07 ± 3.91	3.01 ± 1.68	0.0004
NKT (% of T cells)	8.07 ± 13.25	4.99 ± 5.47	0.263

**Fig. 2:**
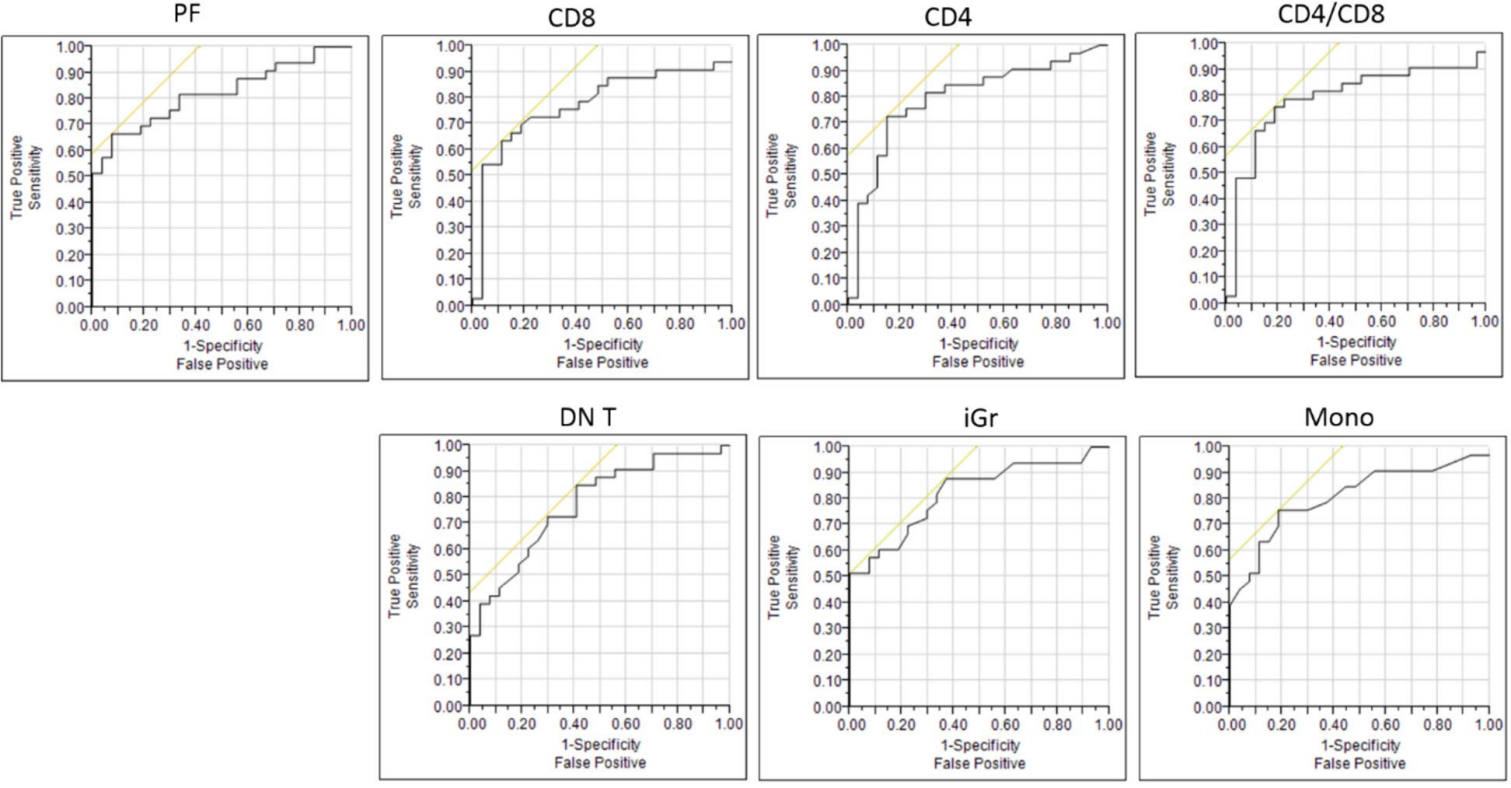
Plots showing curves of ROC analysis for all the parameters that show the most significant differences between aggressive and indolent B-NHLs

To evaluate the potential of DNA content analysis parameters and immune cells subpopulations to differentiate between aggressive and indolent B-NHLs, a ROC analysis was performed for all the parameters that show the most significant differences between the groups. We identified PF > 6.8% as an optimal cutoff value to discriminate between aggressive and indolent B-NHLs with the highest specificity (92.6%). We identified iGr > 0.9% and DN T-cells > 3.1% as optimal cutoff values to discriminate between aggressive and indolent B-NHLs with the highest sensitivity (88% and 82.3% respectively). (Curves of ROC analysis are displayed in Fig. 2) (Optimal cutoffs for all the parameters analyzed by ROC are summarized in Table 2).

**Table 2:** Optimal cutoffs to differentiate between specimens with aggressive and indolent B-NHLs

Parameter	Optimal Cutoff	AUC	% Sensitivity	% Specificity
PF	> 6.8%	0.827	67.6	92.6
CD8 T cells	> 30%	0.783	64.7	89
CD4 T cells	< 70%	0.799	73.5	86
CD4/CD8 ratio	< 2.92	0.796	76.4	82
DN T cells	> 3.1%	0.770	82.3	60
iGr	> 0.9%	0.882	88	37
Mono	> 1.5%	0.817	76.4	81.5

## Discussion

The comparison between aggressive and indolent B-NHLs showed significant increase in the rate of DNA aneuploidy and in the incidences of cells in PF in aggressive B-NHLs. These findings confirm, strengthen, and extend our previous observations in CD10 positive B-NHLs [6], which together with other previous reports [7–10] further suggest the practical utility of DNA content analysis in B-NHLs classification. As was also noted in our previous study, the correlation of PF with the proliferative index determined by ki-67 staining, further indicate PF as a marker of aggressiveness.

As was previously shown, the high rate of DNA aneuploidy in aggressive lymphomas may result from an unequal cell division [11]. Additionally, aggressive lymphoma cells are more likely to be found in a state of arrest in the different cell cycle stages of division without having completed the cell cycle properly [12]. As was previously explained [13], the high incidences of cells in PF in aggressive B-NHLs may be the consequence of accumulation of driver mutations that control cell division. In addition, increased proliferation could be promoted by signals and factors that are imposed and secreted from cells in the tumor microenvironment [14].

Analysis of the surrounding immune cells in our study demonstrated lower percentages of total CD45^+^ cells in aggressive relative to indolent B-NHLs. Stromal cell remodeling was shown to be the landscape of B-NHLs [14]. Interestingly, this single cell atlas of the human lymph node, demonstrated an increase of non-hematopoietic cells such as blood endothelial cells, follicular dendritic cells and marginal reticular cells and a decrease in lymphatic endothelial cells alongside the transformation of FL to DLBCL. As elucidated in this work, increased stromal cells in the LN of DLBCL reflects increased angiogenesis as an important mechanism that facilitates the lymphoma aggressiveness. Although it needs to be confirmed, our observation of lower percentages of CD45^+^ hematopoietic cells in aggressive B-NHLs may be related to a relative increase percentages of non-hematopoietic stromal cells and thus supports this elucidation. However, we could not rule out that this observation is also related to increased cell debris in the aggressive lymphoma samples.

In addition to reduced percentages of CD45^+^ cells, we observed increased percentages of monocytes, mGr and iGr and relatively lower percentages of lymphocytes in aggressive compared to indolent B-NHLs. The high percentages of these cell populations are in line with several reports showing the association of increased tumor infiltrating myeloid derived cells and myeloid suppressor cells within aggressive B-NHLs samples [15, 16]. As was previously demonstrated, these cells can potentially contribute to lymphoma aggressiveness by the production of pro-angiogenic factors that facilitate stromal cell remodeling and angiogenesis [17]. Additionally, as group of tumor-suppressor cells [17], monocytes, iGr and mGr can contribute to disease aggressiveness by the secretion of inhibitory molecules that suppress the immune response and the activity of cytotoxic T-cells against the tumor cells [18].

Corresponding with the increase of myeloid cells, our observations show significant alterations in the percentages of T-cells subsets, including reduced CD4^+^ T cells, and relative increase of CD8^+^ T cells, DP and DN T-cells in aggressive as compared to indolent B-NHLs. The relative reduced percentages of CD4 T-cells and increased CD8 T-cells, are in line with previous report [19]. However, as we did not assessed their functionality, we currently don’t know if the infiltrated CD8 T-cells in our aggressive B-NHLs are immunologically exhausted or functional and could potentially act against the tumor cells when treated with immune-check point inhibitors (Wu et al*.*, 2022). In addition, as in other disease settings, infiltrated CD8 T-cells were shown to promote pathological angiogenesis (Wu et al*.*, 2022), we could not exclude that these cells may also collaborate with non-hematopoietic stromal cells to increase angiogenesis in the microenvironment of aggressive B-NHLs. Parallel to CD8 T-cells, the increase percentages of DN and DP T cells which are known to have autoimmune activity [20] may imply an increased autoimmune response against the tumor cells in aggressive B-NHLs. However, as these cells also have a regulatory role (Collins, Jacks and Pavletich, 1997) we could not exclude that these cells collaborate with myeloid cells to suppress the T-cell response against the tumor cells.

**Fig. 3:**
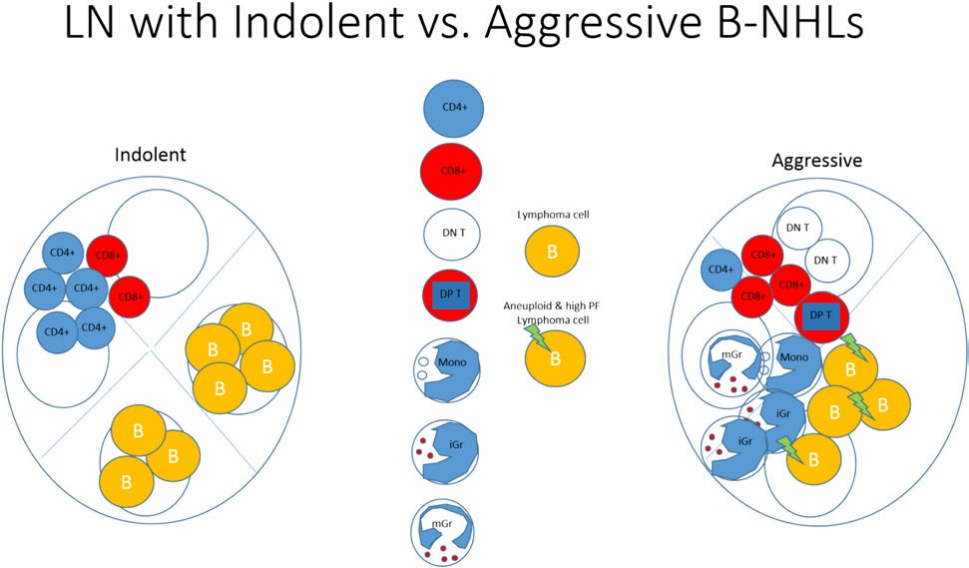
Ilustration showing a summary of our observations. Compared with indolent B-NHLs, aggressive B-NHLs show increased DNA aneuploidy and PF, increased monocytes, immature-granulocytes, mature granulocytes, CD8^+^ T-cells, Double-Negative-T-cells and Double-Positive-T-cells, and decreased lymphocytes and CD4^+^ T-cells

Our study shows potential diagnostic utility for integration of PF and iGr in differentiating specimens with aggressive and indolent B-NHLs by FC, with high specificity and sensitivity. These results support our proposal that surrounding immune cells could be employed in the process of lymphoma diagnosis and classification (Fig. 3). From a clinical standpoint, there is a tendency towards a minimally invasive procedure for diagnosis of lymphoproliferative disorders. While an excisional biopsy may be a reasonable option for superficial lymphadenopathy, it is a more invasive procedure for deep-seated lesions, requiring inpatient surgery under full anesthesia, resulting in longer recovery time and a higher risk of complications than core-needle biopsy.

Aggressive and indolent lymphomas differ in their clinical behavior and require different clinical follow-up and treatment strategies [21]. One of the advantages of FC is that it requires a minimal number of cells that may be achieved even through fine needle aspiration which is even less invasive than core needle biopsy. Therefore, the added value of the combination of DNA content analysis and surrounding immune cell based biomarkers presented in the current study may enable the FC method to become a more informative and accurate diagnostic method for lymphoma diagnosis in minimally invasive procedures.

This study has several limitations. First, the samples in this study include relatively small, non-homogenous representation of indolent lymphomas and lack of other aggressive lymphomas such as Burkitts lymphoma. There is also lack of comparison between MCLs with different clinical disease behaviour, different subtypes of FL and FL transformation to DLBCL. Second, we used manual and expert based gating strategy that is more flexible but exposed to self-biases and individual errors. Third, it was difficult to accurately gate and measure PF in DNA aneuploid samples, and differentiate and measure myeloid immune cells subsets by FC in some specimens, specially of aggressive BNHLs, due to increased necrotic cells, non-specific staining and strong autoflorecence of the lymphoma cells. In this regard, the use of other and more specific markers to better differentiate the myeloid subsets (i.e. monocytes and granulocytes) in the specimens is recommended. In addtion As regard to the lymphoid cells subset with the low incidences, there is an increase chance for mistakes in determination of their level specially in specimens with limited cell numbers. The lack of more detailed functional data regarding the immune cell subsets that were found are also limiting the study.

In conclusion, we have found significant alterations in the rate of DNA aneuploidy and proliferative fraction as well as in the percentages of myeloid and lymphoid immune cells subsets and emphasized their potential diagnostic utility. Further studies with prospective follow-up and correlations to prognostic clinical characteristics as well as the use of functional markers for angiogenesis and immune response will yield information that can be used to predict prognosis and accurate individual treatment for patients.

## Supplementary Information

Below is the link to the electronic supplementary material.

Supplementary file1 (DOCX 14 KB)
