# Supplementary material for: Flow-cytometry Assessment of DNA content and Immunophenotyping of Immune-cells in Lymph-node-specimens as a Potential Diagnostic Signature of Aggressiveness in B-Non-Hodgkin Lymphomas
Source: Ann Hematol. 2024 May 23;103(10):4203–10. doi: 10.1007/s00277-024-05807-8 (PMC11512821; doi:10.1007/s00277-024-05807-8)
Supplement: Supplementary file 1 — Supplementary file1 (DOCX 14 KB) [file 277_2024_5807_MOESM1_ESM.docx]

Supplementary table 1: Classification and prevalence of the specimens with B-NHL

| B-NHL Classification | N (% of total) | Clinical behavior |
| --- | --- | --- |
| DLBCL (Diffused Large B cell Lymphoma) | 30 (48.3%) | Aggressive |
| FL (Follicular Lymphoma) | 18 (29%) | Indolent |
| MZL (Marginal Cell Lymphoma) | 8 (13%) | Indolent |
| MCL (Mantle Cell Lymphoma) | 3 (5%) | Aggressive |
| ALCL (Anaplastic large cell lymphoma) | 1 (1.6 %) | Aggressive |
| SLL (Small Cell Lymphoma) | 2 (3.2 %) | Indolent |
| Total | 62 (100%) | Aggressive: Indolent N (%)  34 : 28 (55:45) |
